# A Study of Dose and Effect in Initiation of Skin Tumours by a Carcinogenic Hydrocarbon

**DOI:** 10.1038/bjc.1960.63

**Published:** 1960-09

**Authors:** J. K. Ball, J. A. McCarter


### A STUDY OF DOSE AND EFFECT IN INITIATION OF SKIN TUMOURS BY A CARCINOGENIC HYDROCARBON

J. K. BALL AND J. A. McCARTER

*From the Department of Biochemistry, Dalhousie University, Halifax, Nova Scotia, Canada*

Received for publication June 29, 1960

IN the study described in this paper, we have attempted to learn how the incidence of tumours elicited in mouse skin by initiating and promoting stimuli (reviewed by Salaman, 1958) is related, quantitatively, to the amount of carcinogenic hydrocarbon absorbed by the skin, rather than to the amount applied.

Methods have been devised (McCarter, 1956) to confine the applied hydrocarbon to a measured area of the skin of an immobilized mouse and to limit the amount absorbed by limiting the time that excess hydrocarbon remains on the skin. Some animals are killed so that the amount of the hydrocarbon absorbed by the skin can be determined. Others are allowed to live and are treated topically with croton oil to produce tumours.

Using these techniques, it was possible to show (McCarter, Szerb and Thompson, 1956) that the number of tumours produced in the skin of a mouse varied directly with the area of skin covered by the hydrocarbon, with the time allowed for absorption and with the logarithm of the concentration of the solution applied (9,10-dimethyl-1,2-benzanthracene in liquid paraffin). Subsequently (McCarter, 1958), an attempt was made to relate the tumour incidence to the amount of hydrocarbon (3,4-benzopyrene) that penetrated unit area of skin in a given time following the application of a solution of the substance in acetone. The present paper describes a similar, but more extensive investigation using 9,10-dimethyl-1,2-benzanthracene, hereafter called DMBA.

### METHODS

### *Application of hydrocarbon to the skin*

The animals used were female mice of the CFW strain (Carworth Farms Inc., New City, New York) 8 to 10 weeks of age. They were housed in groups of 10 in acrylic plastic boxes with stainless steel tops. The bedding was sawdust. Water and Purina Fox Chow Cubes were freely available.

Each animal was immobilized by the intraperitoneal injection of a solution of Meprobamate (Miltown, kindly supplied by Dr. F. M. Berger, Wallace Laboratories, New Brunswick, N.J.) using 0·40 g. per kg. body weight. This was prepared as an 0·8 per cent solution in 5 per cent gum acacia. A second dose of 0·2 g. per kg. body weight was given 4 hours after the first to those animals which were to be immobilized for 7 hours.

Hair was removed from the back using electric clippers. Only those mice were used that were in the resting phase of the hair cycle, as indicated by the lack of a " fuzzy " appearance after clipping and, in those animals kept for tumour production, lack of hair growth during the next few days. This technique might have allowed a few animals early in the growth stage to have been included among those used for the chemical analyses, but we wished to avoid any complications that might arise by prior treatment of the skin, by plucking, for example.

A solution of DMBA in redistilled acetone was applied to a circular area of $2 \cdot 3 \pm 0 \cdot 3$ sq.cm. (Standard Deviation) on the mid-portion of the back using the applicator described by McCarter (1956). The mouse was then placed until required in a dark incubator maintained at 25° C. Then, the excess of hydrocarbon remaining on the skin was carefully removed by washing with diethyl ether (McCarter, 1956). The animal was returned to its cage to await further treatment with croton oil, or was killed so that chemical analysis of the dosed area of skin could be made.

*Production of tumours*

Beginning three weeks after treatment of the animal with DMBA, each mouse was painted twice weekly with 2·5 per cent croton oil (Bush and Co., Canada) in liquid paraffin (paraffin oil, Fisher Scientific Co., Montreal, Canada ; Viscosity 125/135, N.F.) using an artist's No. 6 camel hair brush. The solution was spread over an area greater than that treated with DMBA. The appearance of the back of the mouse was recorded in a sketch once each week (usually checked by another observer) so that the time of appearance and subsequent history of each tumour could be noted. Any tumour larger than 2 mm. in diameter (approximately) and observed on 3 consecutive weeks was recorded.

*Chemical analysis*

The animal was killed by a blow on the head. The position of the circle treated with DMBA was made visible by brief illumination with an ultraviolet light having the major part of its emission at 366 $m\mu$. The treated area of skin was excised together with a narrow untreated margin.

A suitable number of such treated circles (5 for contact-times of 1 hour or longer ; 10 for times less than 1 hour) were pooled and extracted in a Soxhlet apparatus with 95 per cent ethanol (80 ml.) for 24 hours. Under these conditions, extraction of the hydrocarbon from the skin was complete. The ethanol extract was heated under a reflux condenser with 2 ml. of 50 per cent potassium hydroxide for 6 hours to saponify lipids. After cooling, the mixture was diluted with water and extracted repeatedly with petroleum ether (b.p. 30 to 60° C.) in a specially-constructed glass U-tube (so that contamination with grease et cetera could be avoided). The petroleum ether extracts were combined and evaporated to dryness under reduced pressure.

It was necessary to separate the hydrocarbon from substances in the extract that interfered with the measurement of the ultraviolet absorption spectrum. The residue derived from the evaporation of the petroleum ether was dissolved in a mixture of petroleum ether and benzene in the proportions 80 : 20 (v/v) and the solution was transferred to a chromatographic column of Florisil, 9·7 g., 60 to 100 mesh in a tube 2·2 cm. in diameter. (Florisil, Floridin Co., Tallahassee, Florida). The solvent mixture was passed through the column and collected in 10 ml. portions. Those that contained DMBA were located using ultraviolet absorption spectroscopy, pooled and evaporated to dryness under reduced pressure. The residue was dissolved in 95 per cent ethanol and transferred quantitatively to a suitable volumetric flask. The ultraviolet absorption spectrum of the solution was measured relative to that of a similarly prepared control solution derived from a quantity of skin equal to that used in the analysis and obtained from mice dosed on the skin with acetone and washed with diethyl ether. Silica cells having a light path of 1 cm. were used in the Beckman DK-2 Ratio Recording Spectrophotometer. The absorption spectrum from 260 to 340 mμ. provided qualitative identification of DMBA and the concentration was calculated from the molar extinction coefficient at 297 mμ. ($E_{molar}$ at 297 mμ. = 4·90).

Using the analytical procedure described above, amounts of DMBA varying from 0·6 to 6·4 μg. added to mouse skin were recovered as noted in Table I.

TABLE I.—*Analytical Recovery of Amount of DMBA Added to Mouse Skin*

| Added (μg.) | Recovered (μg.) |
|---|---|
| 0·6 | 0·6 |
| 2·2 | 2·2 |
| 3·2 | 3·4 |
| 6·4 | 6·3 |
| 6·4 | 6·4 |
| 6·4 | 6·0 |

### RESULTS

In experiments to determine the amounts of DMBA in the skin after washing the excess from the surface, different results were obtained by different experimenters. The data of Table II illustrate these results. It is apparent that, although agreement between the two sets of results was not achieved, each experimenter could reproduce his own results with accuracy (as indicated by the standard errors). For reasons which are considered in the Discussion, experiments were undertaken to learn if variation in the amounts of hydrocarbon applied to the skin could influence its absorption.

TABLE II.—*Amounts of DMBA in Skin of Mouse at Various Times After Application of Solution* 0·25 *per cent in Acetone. Means of* 4 *Analyses,* 10 *Circles per Analysis for Times Less Than* 1 *Hour ;* 5 *for Times Greater Than* 1 *Hour. Standard Errors. Area,* 2·3 *sq.cm.*

| Time (hours) | Investigation B (μg.) | Investigation H (μg.) |
|---|---|---|
| 0·25 | 1·09±0·05 | 0·35±0·03 |
| 0·5 | 1·05±0·13 | 0·35±0·08 |
| 1 | 1·40±0·04 | 1·47±0·13 |
| 3 | 1·89±0·12 | 2·73±0·25 |
| 5 | 2·09±0·06 | 3·73±0·16 |
| 7 | . . | 4·18±0·06 |

A volume of 0·15 ml. of an acetone solution of DMBA of concentrations 0·005 to 1·0 per cent was applied to an area of 2·3 sq.cm. of the skin of the back of an immobilized mouse (5 in each group) using the procedures described above. Three hours later, the excess of hydrocarbon on the skin was removed and the treated areas were excised, pooled and analyzed for their content of DMBA. The results of this experiment are recorded in Table III. It is apparent that the

TABLE III.—*Influence of Variation in Amount of DMBA Applied to Skin on Amount that Penetrates in 3 Hours. Means of 4 Analyses, 5 Circles per Analysis.* 0·15 *ml. Applied to* 2·3 *sq.cm. Standard Errors.*

| Concentration of solution (%) | DMBA applied ($\mu$g.) | DMBA in skin ($\mu$g.) |
|---|---|---|
| 0·005 | 7·5 | 0·50±0·07 |
| 0·01 | 15 | 0·81±0·18 |
| 0·05 | 75 | 3·48±0·09 |
| 0·10 | 150 | 2·44±0·05 |
| 0·20 | 300 | 1·87±0·03 |
| 0·30 | 400 | 1·38±0·20 |
| 0·70 | 1050 | 1·55±0·10 |
| 1·0 | 1500 | 1·88±0·21 |

absorption of DMBA increased as the amount applied was increased from 7·5 to 75 $\mu$g., then decreased and became relatively constant when 300 to 1500 $\mu$g. was applied.

When an amount of 75 $\mu$g. DMBA was applied to the skin in 0·15 ml. of 0·05 per cent solution in acetone and the excess hydrocarbon was washed off at various times thereafter, the results shown in Table IV were obtained.

TABLE IV.—*The Penetration of DMBA into the Skin After the Application of* 75 $\mu$*g.* (0·15 *ml. of* 0·05 *per cent Solution in Acetone*) *to* 2·3 *sq.cm. Means of* 3 *Analyses,* 5 *Circles per Analysis. Standard Errors.*

| Time (hours) | DMBA in skin ($\mu$g.) |
|---|---|
| 0·25 | 0·15±0·03 |
| 0·50 | 0·41±0·05 |
| 1 | 0·89±0·07 |
| 2 | 1·91±0·13 |
| 3 | 3·48±0·09 |
| 5 | 4·74±0·15 |

*Tumour incidence*

The data for the distribution of tumours among all the mice exposed to DMBA are recorded in Table V. Also included in this table are the results obtained using a control group of mice dosed with acetone, immobilized for 7 hours and washed with diethyl ether. These mice, like those that had been treated with DMBA, were painted twice weekly with croton oil. Data for the time to appearance of the first tumour in a mouse (timed from the day that treatment with croton oil was begun) are recorded in Table VI. Times to appearance of all the tumours are reported in Table VII. A summary of all the data is given in Table VIII. Fig. 1 and 2 are discussed in the next section.

### DISCUSSION

Before undertaking this work, we thought that variation in the amount of solid hydrocarbon applied to the skin, such as could be achieved by applying

TABLE V.—*Distribution of Tumours Among Mice*

| Concentration DMBA (%) | Exposure (hours) | DMBA in skin (μg.) | Number of mice having following number of tumours: 0 | 1 | 2 | 3 | 4 | 5 | 6 | 7 | 8 | 9 | 10 | 11 | 12 | 13 | 14 | 15 | Number of mice | Number of tumours | Tumours per mouse | Standard deviation |
|---|---|---|---|---|---|---|---|---|---|---|---|---|---|---|---|---|---|---|---|---|---|---|
| *Zero* | — | — | 83 | 6 | 1 | .. | .. | .. | .. | .. | .. | .. | .. | .. | .. | .. | .. | .. | 90 | 8 | 0·09 | .. |
| 0·05 | 0·25 | 0·15 | 22 | 14 | 9 | 6 | 1 | .. | 2 | .. | .. | 1 | .. | .. | .. | .. | .. | .. | 55 | 75 | 1·37 | 1·78 |
| 75 μg. applied | 0·50 | 0·41 | 26 | 8 | 6 | 7 | 4 | 1 | 3 | 1 | .. | .. | 1 | .. | .. | .. | .. | .. | 57 | 97 | 1·70 | 2·22 |
| | 1 | 0·89 | 14 | 11 | 8 | 4 | 5 | 3 | 4 | 2 | 2 | .. | 1 | 1 | .. | .. | .. | .. | 55 | 149 | 2·71 | 2·79 |
| | 3 | 3·48 | 19 | 9 | 8 | 4 | 7 | 4 | 2 | 1 | 1 | 2 | .. | 1 | .. | .. | .. | .. | 58 | 141 | 2·43 | 2·69 |
| | 5 | 4·74 | 16 | 11 | 5 | 4 | .. | 10 | 2 | 1 | 1 | 1 | 2 | .. | .. | 1 | 1 | .. | 55 | 166 | 3·02 | 3·46 |
| 0·25 | 0·25 | 1·09 | 27 | 6 | 4 | 4 | .. | 3 | 4 | 2 | .. | .. | 3 | .. | .. | .. | .. | .. | 56 | 133 | 2·38 | 3·16 |
| B 300 μg. applied | 0·50 | 1·05 | 10 | 8 | 10 | 6 | 9 | 5 | 2 | 2 | 1 | 2 | .. | .. | 1 | .. | .. | .. | 56 | 171 | 3·05 | 2·65 |
| | 1 | 1·40 | 18 | 8 | 5 | 3 | 6 | 1 | 4 | 1 | 2 | 1 | 1 | .. | .. | .. | .. | .. | 50 | 122 | 2·44 | 2·78 |
| | 3 | 1·89 | 24 | 8 | 4 | 3 | 5 | 4 | .. | 1 | 2 | 1 | .. | 2 | 1 | 2 | .. | .. | 57 | 157 | 2·76 | 3·73 |
| | 5 | 2·09 | 24 | 9 | 9 | 5 | 3 | 2 | .. | .. | 2 | .. | .. | 2 | .. | 1 | 1 | .. | 58 | 129 | 2·23 | 3·33 |
| 0·25 | 0·25 | 0·35 | 29 | 9 | 5 | 4 | 3 | 1 | 2 | .. | 2 | 1 | .. | .. | .. | .. | .. | .. | 56 | 85 | 1·52 | 2·29 |
| H 100–150 μg. applied | 0·50 | 0·35 | 16 | 15 | 9 | 3 | 5 | 2 | 2 | .. | 2 | .. | .. | .. | .. | .. | .. | .. | 54 | 100 | 1·86 | 2·04 |
| | 1 | 1·47 | 25 | 10 | 11 | 8 | 7 | 3 | 2 | 1 | 1 | .. | 2 | 3 | .. | .. | .. | .. | 73 | 179 | 2·49 | 2·92 |
| | 3 | 2·73 | 27 | 8 | 9 | 6 | 12 | 4 | 6 | 1 | 1 | .. | .. | .. | .. | .. | .. | .. | 74 | 163 | 2·24 | 2·17 |
| | 5 | 3·73 | 5 | 4 | 2 | 9 | 3 | 3 | 2 | 4 | 2 | .. | .. | .. | .. | .. | .. | .. | 34 | 118 | 3·48 | 2·41 |
| | 7 | 4·18 | 6 | 3 | 4 | 5 | 5 | 8 | 3 | 2 | 2 | .. | .. | .. | .. | .. | .. | 1 | 39 | 149 | 3·80 | 2·96 |

TABLE VI.—*Time to Appearance of First Tumour in a Mouse Dated from Time Treatment with Croton Oil was Begun*

| Concentration DMBA (%) | Exposure (hours) | DMBA in skin (μg.) | Number of mice bearing tumours in following number of weeks: 3 | 4 | 5 | 6 | 7 | 8 | 9 | 10 | 11 | 12 | 13 | 14 | 15 | 16 | 17 | 18 | 19 | 20 | Total tumour bearers | Total mice |
|---|---|---|---|---|---|---|---|---|---|---|---|---|---|---|---|---|---|---|---|---|---|---|
| 0·05 | 0·25 | 0·15 | .. | .. | .. | .. | 2 | 4 | 9 | 7 | 3 | .. | 1 | 1 | 2 | .. | 1 | 3 | .. | .. | 33 | 55 |
| | 0·50 | 0·41 | .. | .. | .. | .. | 2 | 5 | 8 | 5 | 3 | 1 | 2 | 3 | 1 | .. | 1 | .. | .. | .. | 31 | 57 |
| | 1·0 | 0·89 | .. | .. | .. | 1 | 6 | 5 | 9 | 3 | 7 | 4 | 2 | .. | 1 | 2 | 1 | .. | .. | .. | 41 | 55 |
| | 3·0 | 3·48 | .. | .. | .. | .. | 4 | 2 | 9 | 8 | 1 | 4 | 1 | 7 | 1 | 1 | 1 | .. | .. | .. | 39 | 58 |
| | 5·0 | 4·75 | .. | .. | .. | 9 | 2 | 10 | 5 | 5 | 1 | 3 | 4 | .. | .. | .. | .. | .. | .. | .. | 39 | 55 |
| 0·25 B | 0·25 | 1·09 | .. | .. | .. | 1 | 2 | 6 | 8 | 3 | 1 | 3 | 2 | 2 | 1 | .. | .. | .. | .. | .. | 29 | 56 |
| | 0·50 | 1·05 | .. | .. | .. | .. | 4 | 9 | 15 | 7 | 2 | 6 | 1 | 1 | .. | .. | .. | 1 | .. | .. | 46 | 56 |
| | 1·0 | 1·4 | .. | .. | .. | 3 | .. | 4 | 11 | 5 | 2 | 2 | 2 | .. | 2 | 1 | .. | .. | .. | .. | 32 | 50 |
| | 3·0 | 1·89 | .. | .. | .. | 1 | 4 | 4 | 7 | 4 | 4 | 6 | 2 | .. | .. | .. | .. | .. | .. | 1 | 33 | 57 |
| | 5·0 | 2·09 | .. | .. | .. | 1 | 5 | 6 | 9 | 6 | 1 | 2 | 1 | 1 | 1 | 1 | .. | .. | .. | .. | 34 | 58 |
| | 7·0 | .. | .. | 1 | .. | 1 | 5 | 4 | 3 | 3 | 1 | 2 | .. | 1 | 1 | .. | .. | 2 | .. | .. | 24 | 33 |
| 0·25 H | 0·25 | 0·35 | .. | .. | 7 | 1 | 4 | 4 | 2 | 3 | 1 | 2 | 1 | 1 | 1 | .. | .. | .. | .. | .. | 27 | 56 |
| | 0·50 | 0·35 | 1 | .. | .. | 8 | 4 | 6 | 2 | 2 | 2 | 3 | 4 | 1 | 1 | .. | 2 | 2 | .. | .. | 38 | 54 |
| | 1·0 | 1·47 | .. | .. | .. | 1 | 6 | 8 | 8 | 4 | 8 | 2 | 2 | 2 | 1 | 3 | 1 | .. | .. | .. | 46 | 73 |
| | 3·0 | 2·73 | .. | .. | .. | 3 | 7 | 13 | 13 | 2 | 4 | 1 | 2 | 2 | .. | .. | .. | .. | .. | .. | 47 | 74 |
| | 5·0 | 3·73 | .. | .. | .. | .. | 5 | 10 | 6 | 1 | 2 | 4 | .. | .. | .. | .. | .. | 1 | .. | .. | 29 | 34 |
| | 7·0 | 4·18 | .. | .. | .. | 3 | 3 | 8 | 10 | 1 | 2 | 1 | 1 | 1 | 1 | 1 | .. | 1 | .. | .. | 33 | 39 |

TABLE VII.—*Time to Appearance of All Tumours Dated from Time Treatment with Croton Oil was Begun*

| Concentration DMBA (%) | Exposure (hours) | DMBA in skin (μg.) | Number of tumours recorded in following weeks: 3 | 4 | 5 | 6 | 7 | 8 | 9 | 10 | 11 | 12 | 13 | 14 | 15 | 16 | 17 | 18 | 19 | 20 | Total tumours |
|---|---|---|---|---|---|---|---|---|---|---|---|---|---|---|---|---|---|---|---|---|---|
| 0·05 | 0·25 | 0·15 | .. | .. | .. | .. | 3 | 8 | 11 | 14 | 7 | 3 | 3 | 5 | 3 | 7 | 7 | 4 | .. | .. | 75 |
| | 0·50 | 0·41 | .. | .. | .. | .. | 2 | 10 | 17 | 12 | 12 | 11 | 7 | 7 | 6 | 1 | 5 | 3 | .. | 4 | 97 |
| | 1·0 | 0·89 | .. | .. | .. | 1 | 9 | 7 | 28 | 16 | 16 | 25 | 11 | 8 | 9 | 11 | 4 | 1 | 2 | 1 | 149 |
| | 3·0 | 3·48 | .. | .. | .. | .. | 9 | 4 | 19 | 19 | 7 | 18 | 14 | 23 | 12 | 8 | 4 | 2 | 2 | .. | 141 |
| | 5·0 | 4·74 | .. | .. | .. | 17 | 19 | 28 | 16 | 37 | 11 | 21 | 7 | 3 | 2 | 5 | .. | .. | .. | .. | 166 |
| 0·25 B | 0·25 | 1·09 | .. | .. | .. | 1 | 6 | 18 | 30 | 24 | 9 | 10 | 12 | 4 | 3 | 8 | 2 | 3 | 1 | 2 | 133 |
| | 0·50 | 1·05 | .. | .. | .. | .. | 8 | 23 | 38 | 21 | 20 | 27 | 7 | 7 | 7 | 10 | 1 | 2 | .. | .. | 171 |
| | 1·0 | 1·40 | .. | .. | .. | 6 | 3 | 9 | 25 | 22 | 9 | 10 | 8 | 4 | 12 | 7 | 3 | 3 | .. | 1 | 122 |
| | 3·0 | 1·89 | .. | .. | .. | 2 | 9 | 5 | 47 | 23 | 10 | 22 | 14 | 1 | 7 | 9 | 3 | 1 | 1 | 3 | 157 |
| | 5·0 | 2·09 | .. | .. | .. | 1 | 7 | 10 | 37 | 17 | 15 | 17 | 6 | 9 | 3 | 3 | 1 | 2 | .. | 1 | 129 |
| | 7·0 | .. | .. | 1 | .. | 1 | 14 | 4 | 12 | 8 | 3 | 27 | 5 | 7 | 3 | 6 | .. | 4 | 2 | 1 | 98 |
| 0·25 H | 0·25 | 0·35 | .. | .. | 9 | 8 | 13 | 13 | 8 | 6 | 8 | 3 | 6 | 4 | 4 | 2 | .. | .. | .. | 1 | 85 |
| | 0·50 | 0·35 | 1 | .. | .. | 9 | 11 | 12 | 12 | 7 | 6 | 6 | 12 | 4 | 7 | 5 | 3 | 5 | .. | .. | 100 |
| | 1·0 | 1·47 | .. | .. | .. | 1 | 9 | 15 | 37 | 23 | 27 | 20 | 16 | 13 | 9 | 6 | 1 | 2 | .. | .. | 179 |
| | 3·0 | 2·73 | .. | .. | .. | 3 | 13 | 25 | 42 | 25 | 14 | 15 | 5 | 8 | 7 | 2 | 2 | 2 | .. | .. | 163 |
| | 5·0 | 3·73 | .. | .. | .. | .. | 10 | 23 | 22 | 8 | 13 | 15 | 9 | 4 | 5 | 6 | .. | 3 | .. | .. | 118 |
| | 7·0 | 4·18 | .. | .. | .. | 4 | 18 | 24 | 24 | 6 | 20 | 12 | 5 | 19 | 6 | 4 | 4 | 3 | .. | .. | 149 |

TABLE VIII.—*Data of Tables II and IV to VII Rearranged to show Variation in Tumour Incidence and Latent Period with Variation in DMBA in Skin. Standard Errors are shown.*

| DMBA in skin ($\mu$g.) | Tumours per mouse | Number of mice | Latent period: First tumour (weeks) | Latent period: All tumours (weeks) |
|---|---|---|---|---|
| 0·00 | 0·09 | 90 | .. | .. |
| 0·15±0·03 | 1·36 | 55 | 10·85±0·57 | 11·92±0·39 |
| 0·35±0·03 | 1·52 | 56 | 8·37±0·57 | 9·29±0·35 |
| 0·35±0·08 | 1·86 | 54 | 9·84±0·63 | 10·84±0·34 |
| 0·41±0·05 | 1·70 | 57 | 10·35±0·45 | 11·80±0·36 |
| 0·89±0·07 | 2·71 | 55 | 10·09±0·42 | 11·56±0·40 |
| 1·05±0·13 | 3·06 | 56 | 9·45±0·31 | 10·78±0·18 |
| 1·09±0·05 | 2·38 | 56 | 9·86±0·43 | 10·92±0·26 |
| 1·40±0·04 | 2·44 | 50 | 9·91±0·44 | 11·23±0·28 |
| 1·47±0·13 | 2·49 | 73 | 10·32±0·42 | 11·07±0·21 |
| 1·89±0·12 | 2·76 | 57 | 10·03±0·46 | 11·08±0·23 |
| 2·09±0·06 | 2·23 | 58 | 9·89±0·42 | 10·67±0·23 |
| 2·73±0·25 | 2·23 | 74 | 8·87±0·29 | 11·05±0·34 |
| 3·48±0·09 | 2·43 | 58 | 10·85±0·43 | 12·07±0·24 |
| 3·73±0·16 | 3·47 | 34 | 9·28±0·44 | 10·61±0·26 |
| 4·18±0·06 | 3·82 | 39 | 9·48±0·50 | 10·66±0·17 |
| 4·74±0·15 | 3·02 | 55 | 8·77±0·36 | 11·05±0·34 |

varying volumes or concentrations of a solution in a solvent that readily evaporates, should be without effect on the amount of the hydrocarbon absorbed by unit area in a given time, provided that enough was applied to maintain a saturated solution in the sebum. The latter condition should ensure the absorption of the hydrocarbon at the maximal rate. On this basis, the increased absorption of DMBA which resulted when the amount applied was increased from 7·5 to 75 $\mu$g. (Table III) may be explained by assuming that amounts less than 75 $\mu$g. were not enough to provide a saturated solution in the sebum. A different explanation has to be sought for the fact that the absorption decreased as the amount applied was increased from 75 to 300 $\mu$g. and thereafter became relatively constant. It seems probable that, as the bulk of the solid hydrocarbon was increased, it soaked up some of the sebum, particularly that readily available on the surface of the skin, as indeed any powdery substance should do. The importance of sebum for the absorption of DMBA was demonstrated in an experiment in which the skin was washed with diethyl ether before the hydrocarbon was applied. Under these conditions, only 0·56 $\mu$g. of DMBA was absorbed in 2 hours as compared with 1·91 $\mu$g. in the same time by unwashed skin.

The differences obtained by the two experimenters who measured the penetration of DMBA at various times (Table II) must be attributed to the fact that the quantities applied to the skin were different in the two experiments. One investigator (B) applied approximately 300 $\mu$g. or more ; the other (H) applied only 100 to 150 $\mu$g., both in the belief (supported by our earlier experience with 3,4-benzopyrene—McCarter, 1956) that variation in the amount applied was without influence on the amount absorbed. The values recorded in Table II for 3 hours of penetration are consistent with predictions made on the basis of the amounts applied and the data of Table III. However, the data of Tables II and IV show that a greater *initial* penetration of DMBA took place when the amount applied was increased. Thus, in 0·25 hours, 0·15 $\mu$g. of 75, 0·35 of 100–150, and

FIG. 1.—Tumours per mouse plotted against the logarithm of the amount of DMBA in the skin. The straight line fitted by the method of least squares has the equation $Y = 1 \cdot 13 + 1 \cdot 12 \log (10 \times \mu g.\ \text{DMBA})$. Standard error of the slope = 0·21.

FIG. 2.—The number of new tumours observed in each week following the start of treatment with croton oil, calculated by summing the appropriate columns of Table VII.

1·09 of 300 or more had entered the skin. The factors governing the absorption of DMBA by mouse skin must be more complex than we have supposed them to be.

The data of Table IV were obtained having regard for all the known variables. The data are fitted by the straight line $Y = 0{\cdot}004 + 0{\cdot}96$ (hours of contact) where $Y = \mu$g. of DMBA per dosed circle (2·3 sq.cm.). The standard error of the slope was 0·01. The fact that the rate of absorption of the hydrocarbon was constant during the 5 hours' period under consideration suggests that there was no marked alteration in permeability of the skin during that time.

The mistake made in assuming that variation in the amount applied would have no influence on the amount absorbed, would have made it impossible to compare the data on tumour incidence obtained by the two investigators, and certainly it would not have been possible to combine the results as has been done in Table VIII, but for the fact that the amount of DMBA in the skin was measured, thus providing a common basis for comparison. It was important to know that each set of experiments had been performed with a high degree of reproducibility (standard errors, Table III) so that reliance could be placed in the corresponding analytical and tumour data. It was possible, therefore, to use all of the data.

The information provided by these experiments was examined for the existence of relationships between the amount of hydrocarbon in the skin (disregarding the time for absorption as a factor) and the tumour yield. For each group that had received a given dose two measures were available ; one was the average number of tumours borne by the animals that survived the experiment : the other was the proportion of tumour-bearing mice relative to the number of survivors. It must be stressed that the number of tumours recorded per mouse was the number of new tumours produced during the whole period of 20 weeks and was not the incidence at any particular time. The index we have used is independent of the balance struck between the rate of appearance of new tumours and the rate of disappearance of old (discussed by Salaman, 1958). We have not tried to do any calculations relating the rate of regression of tumours to the dose of DMBA (the amount in the skin) because we are uncertain about how to score a tumour as one regressing or having regressed.

The amount of hydrocarbon found in the skin by analysis is less than that which has penetrated because some is metabolized or transported from the site. The rate of loss of DMBA left after washing off the excess and measured over the first twelve hour period following absorption, has a half-life time of the order of 10 hours (Huh and McCarter, 1960). This value may be used to estimate, for example, that during the 7 hour period at the end of which 4·74 $\mu$g. DMBA was present in the skin, 6·2 must have been absorbed, of which 1·4 had been metabolized or transported. This value is very much smaller than that estimated by Booth and Boutwell (1959) who attributed the difference between the amounts of DMBA applied to the skin and recovered later at the site of application in restrained animals, to absorption through the skin. The rates of accumulation and disappearance measured by us are not consistent with the assumption made by these authors. The correction for loss during absorption was not applied by us in assessing the dose-response relationships.

The data of Table VI were analyzed using the method of probits as described by Finney (1950). There was no evidence that the results could not be described by a straight line having the equation $Y = 5{\cdot}17 + 0{\cdot}18 \log (10 \times \mu\text{g. DMBA in skin})$

where $Y$ = probits of response. The variance of the slope was 0·11 ; thus it could not be decided whether or not there was a dependence of tumour yield on log dose.

The data of Table VIII show that an increase in the amount of DMBA from 0 to 0·15 ± 0·03 $\mu$g. was accompanied by an increase in the number of tumours produced per mouse by a factor of 15, whereas a further increase to 4·74 ± 0·15 $\mu$g. (factor of 30, or factor of 40 if the value is corrected for metabolic loss) only doubled the tumour yield. An analysis of variance of the data for the effect of variation in log dose on the number of tumours produced per mouse was carried out according to Snedecor (1946). There was a highly significant ($P < 0{\cdot}001$) regression of tumour yield on the logarithm of the amount of DMBA in the skin. The experimental results are described by the equation Tumours per mouse = 1·13 + 1·12 log (10 × $\mu$g. DMBA in skin). The standard error of the slope was 0·21. It would be incorrect to assume either that this relationship is the only one capable of describing the data or that it can be used to extrapolate much beyond the limits of the data. One cannot decide whether or not the tumour yield continues to increase or if it approaches some limiting value as the amount of DMBA is extrapolated to higher values, though it seems apparent that the response is virtually saturated for amounts of DMBA in the skin greater than 1 or 2 $\mu$g. per dosed circle (about 0·5 to 1 $\mu$g. per sq.cm.). We have no explanation to offer for this apparent approach to saturation unless, perhaps, this observation represents the early onset of the phenomenon recorded by Shubik and Ritchie (1953) as a failure to obtain summation of effects of multiple doses of DMBA. Similarly, it is not possible to decide if there is, or is not, a threshold dose for the initiation of tumours, though it seems clear that if such a dose exists it must be very small indeed.

If the data of the groups (rows) of Table V are plotted with numbers of animals as ordinates and numbers of tumours borne by them (0, 1, 2, 3, etc.) as abscissae, it is learned that the distributions are J-shaped. It might have been expected that the data should conform to a Poisson distribution, but there are very significant deviations of the observed values from the expected, particularly for animals having no tumours and for those having many tumours. In both instances, the numbers observed far exceed the numbers predicted. We attempted to analyze our data by the methods described by Polissar and Shimkin (1954) but without conclusive result. The range of responses observed by us (1·36 tumours per mouse with the lowest dose and 3·02 with the highest) was too narrow to permit a trend to be seen when the standard deviations were plotted against the means. For every group, the observed standard deviation was greater than the calculated (S.D. = $m^{\frac{1}{2}}$ for a Poisson) but it was not possible to obtain evidence for or against the alternative hypotheses that the deviations from a Poisson distribution were due to $A$, heterogeneity of susceptibility among the animals, or $B$, interaction between the tumours. The CFW mouse is not inbred, so that hypothesis $A$ seems a logical explanation of our results. On the other hand, it is conceivable, in the present state of our ignorance, that conditions that allow one tumour to appear in a mouse, might allow other tumours to appear with a greater frequency than would be predicted on a random basis.

If DMBA applied to the skin is left on, instead of being washed off as in our experiments, a single dose may lead to epilation and necrosis of the treated skin (Orr, 1938) and may result in the production of tumours without further treatment (Englebreth-Holm and Iversen, 1951). In our experiments, 31 CFW female mice that had, initially, 4·2 μg. of DMBA in the skin failed to develop any tumours in the subsequent 20 weeks' period during which they were not treated with croton oil. We have not observed in our animals any loss of hair at the treated site in the three weeks' interval between the time of the limited exposure to DMBA and the start of treatment with croton oil. On the other hand, when we left the hydrocarbon on the skin, epilation and necrosis were always observed. More detailed investigation is needed, but these observations suggest that initiation of tumours by DMBA may be achieved with a minute amount of hydrocarbon that does not produce apparent injury to the skin ; injury results from the penetration of a larger quantity of the hydrocarbon, but the number of tumours initiated is not thereby necessarily increased. This is not to imply that injury is not important in the overall process of tumour production, but that for DMBA applied to skin, as for urethane (Roe and Salaman, 1954) and triethylene melamine (Roe and Salaman, 1955) and for DMBA by mouth (Berenblum and Haran-Ghera, 1957) initiation of skin tumours may be dissociated from injury.

Altogether, 2510 tumours of the skin were recorded in this study. Judged by their appearance, growth-rate and invasiveness (or lack of it), only 2 were malignant and the remainder were papillomas. Of 655 tumours in 280 mice treated with 75 μg. DMBA applied to 2·3 sq.cm. only 18 (2·7 per cent) arose outside this area. These were situated on the flanks or near the base of the tail but were always close to the treated circle. Contamination of the skin around this area with a minute amount of DMBA during the washing procedure could account for the distribution of these tumours. The chance of contaminating the skin was greater when the applied quantity of DMBA was greater. Of 1855 tumours produced in 638 mice dosed with 100 to 300 μg. 235, or 12 per cent arose outside, but near, the treated area. Tumours that were obviously outside the treated areas were neglected in compiling Tables V to VIII.

A possible source of error in these experiments could arise from tumours growing close together so that they might be indistinguishable from one another. Tumours were first recognised when they had an area of approximately 1 sq.mm. or about 0·5 per cent of the area in which they could appear. Even in the groups having the highest tumour incidence, tumour-bearing animals had an average of 4·3 each, with a maximum of 15 so that the area occupied when the tumours were first recognisable was a negligible proportion of the total available. Growth of the early-appearing tumours might have interfered with the recording of those appearing late but this could only have been a problem in the animals bearing a large number of tumours, for example, 10 or more. Such animals accounted for only 14 per cent of all tumours produced. Furthermore, most of the tumours (80 per cent) appeared within a period of time (7th to 13th weeks) when all were small and least likely to overgrow one another. It is not likely, therefore, that inability to distinguish tumours was a major source of error in these experiments.

The latent period, dated from the time treatment with croton oil was begun to the time of appearance of the first tumour in a mouse, or averaged for all the tumours appearing in a mouse, did not change in a systematic way with variation in the amount of DMBA in the skin. Inspection of Table VII and Fig. 2 might suggest that groups of tumours arose characterized by different latent periods, indicating perhaps that the tumours were of more than one sort, and lending support to the suggestion made by Shubik, Baserga and Ritchie (1953) that tumours produced by initiating and promoting stimuli in mouse skin differ over a range of growth potential. There is, however, no reason to suppose that the fluctuations we have observed are other than random variations due to chance. Perhaps real differences would have been observed had the animals been kept for a longer time, but we decided to stop the experiment when, as shown in Fig. 2, the tumours stopped appearing.

The methods described in this paper should be capable of furnishing estimates of the relative initiating potencies of different hydrocarbons. We have found by analysis that a unit area of CFW mouse skin absorbs in 1 hour $1 \cdot 4 \times 10^{-9}$ moles of 3,4-benzopyrene and $5 \cdot 5 \times 10^{-9}$ moles of DMBA, after application of acetone solutions of the hydrocarbons. The corresponding tumour incidences were 0·3 tumours per mouse for 3,4-benzopyrene and 2·44 per mouse for DMBA. Was the number induced by DMBA greater than that induced by 3,4-benzopyrene merely because there was more DMBA in the skin? The question cannot be answered without knowing more about the dose-response relationships for the two hydrocarbons.

Earlier work with 3,4-benzopyrene (McCarter, 1958) in mice of the strain I, suggested a pattern of dose and response similar to that recorded here. An attempt to use 3,4-benzopyrene in the CFW mouse resulted in the production of too few tumours to allow conclusions to be drawn with confidence, but the data are approximately described by the equation, Tumours per mouse $= 0 \cdot 25 + 0 \cdot 04$ (moles $\times 10^{-9}$ BP/sq.cm.). The rate of penetration of benzopyrene into CFW skin did not differ significantly from that in I skin. These data allow a rough estimate to be made of the relative sensitivities of the two strains of mouse. For example, $2 \times 10^{-9}$ moles of 3,4-benzopyrene per sq.cm. produced 1·8 tumours per mouse in strain I compared with 0·3 in strain CFW. The same dose of DMBA produced 1·9 tumours per CFW mouse. Apparently, the strain I mouse is about 6 times as sensitive as the CFW, and DMBA is 6 or 7 times as potent an initiating agent as 3,4-benzopyrene. However, these estimates are only approximate because in the CFW mouse, the only parts of the dose-response curves for the two hydrocarbons that overlap involve the highest doses of 3,4-bezopyrene and the lowest of DMBA ; no point was obtained at which the tumour yields were identical and it is not possible to decide if the ratio of responses is independent of dose. One would wish to compare the curves over the regions where response is most markedly dependent on dose rather than where virtual saturation of the biological effect is achieved but this would involve one in the measurement of smaller doses and responses with larger errors than were encountered in the present work. Had the present experiments been done on a smaller scale or with less rigorous control of the variables concerned, it is probable that we should not have been able to draw even the limited conclusions that we have drawn about the nature of the dose-response relationships.

### SUMMARY

1. 9,10-Dimethyl-1,2-benzanthracene (DMBA) was applied in acetone to 2·3 sq.cm. of the skin of immobilized mice. After a time, the skin was washed and some of the treated animals were killed for analysis of the DMBA content of the skin ; others were allowed to live and were treated topically with croton oil to produce tumours.

2. The amount of DMBA entering the skin was dependent on the amount applied and on the time allowed for absorption.

3. The number of tumours borne per mouse was a linear function of the logarithm of the amount of DMBA in the skin.

4. Animals bearing no tumours, and those bearing many, were more numerous than predicted by a Poisson distribution.

5. The time from the start of treatment with croton oil to the appearance of the first tumour, or averaged for all the tumours in a group, appeared to be independent of the amount of hydrocarbon in the skin.

6. Difficulties in the way of obtaining relative potency ratios of different hydrocarbons are discussed.

The authors wish to thank Mr. T. Y. Huh for technical assistance and Dr. N. M. Blackett for helpful discussion. We are grateful to the National Cancer Institute of Canada for a grant of funds.

### REFERENCES

BERENBLUM, I. AND HARAN-GHERA, N.—(1957) *Brit. J. Cancer*, **11**, 85.
*Idem* AND SHUBIK, P.—(1947) *Ibid.*, **1**, 383.
BOOTH, B. A. AND BOUTWELL, R. K.—(1959) *Cancer Res.*, **19**, 79.
ENGELBRETH-HOLM, J. AND IVERSEN, S.—(1951) *Acta path. microbiol. scand.*, **29**, 77.
FINNEY, D. J.—(1950) in ' Biological Standardization '. J. H. Burn, D. J. Finney and L. G. Goodwin, 2nd ed. London (Oxford University Press).
HUH, T. Y. AND McCARTER, J. A.—(1960) *Brit. J. Cancer*, **14**, 591.
McCARTER, J. A.—(1956) *J. nat. Cancer Inst.*, **17**, 399.—(1958) *Acta Un. int. Cancr.*, **15**, 178.
*Idem*, SZERB, J. C. AND THOMPSON, G. E.—(1956) *J. nat. Cancer Inst.*, **17**, 405.
ORR, J. W.—(1938) *J. Path. Bact.*, **47**, 495.
POLISSAR, M. J. AND SHIMKIN, M. B.—(1954) *J. nat. Cancer Inst.*, **15**, 377.
ROE, F. J. C. AND SALAMAN, M. H.—(1954) *Brit. J. Cancer*, **8**, 666.—(1955) *Ibid.*, **9**, 177.
SALAMAN, M. H.—(1958) *Brit. med. Bull.*, **14**, 116.
SHUBIK, P., BASERGA, R. AND RITCHIE, A. C.—(1953) *Brit. J. Cancer*, **7**, 342.
*Idem* AND RITCHIE, A. C.—(1953) *Cancer Res.*, **13**, 343.
SNEDECOR, G. W.—(1946) ' Statistical Methods Applied to Experiments in Agriculture and Biology '. 4th ed., Ames, Iowa (The Collegiate Press, Inc.).


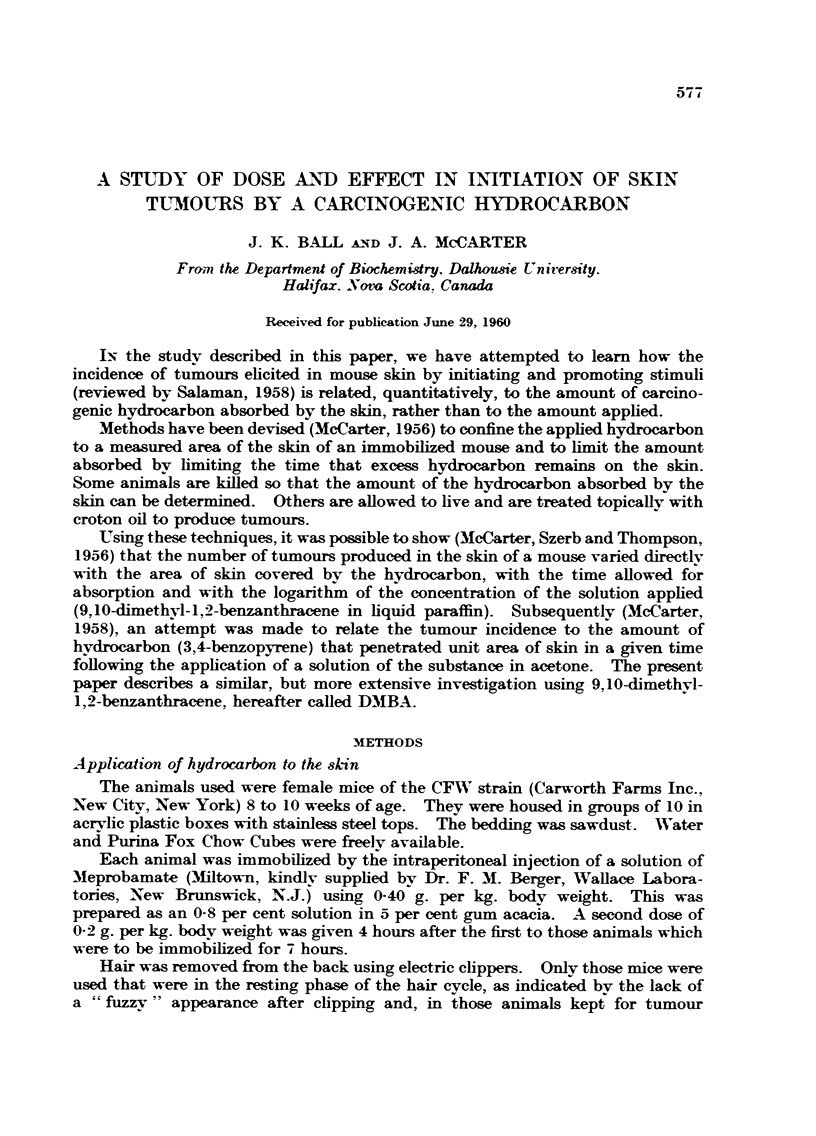

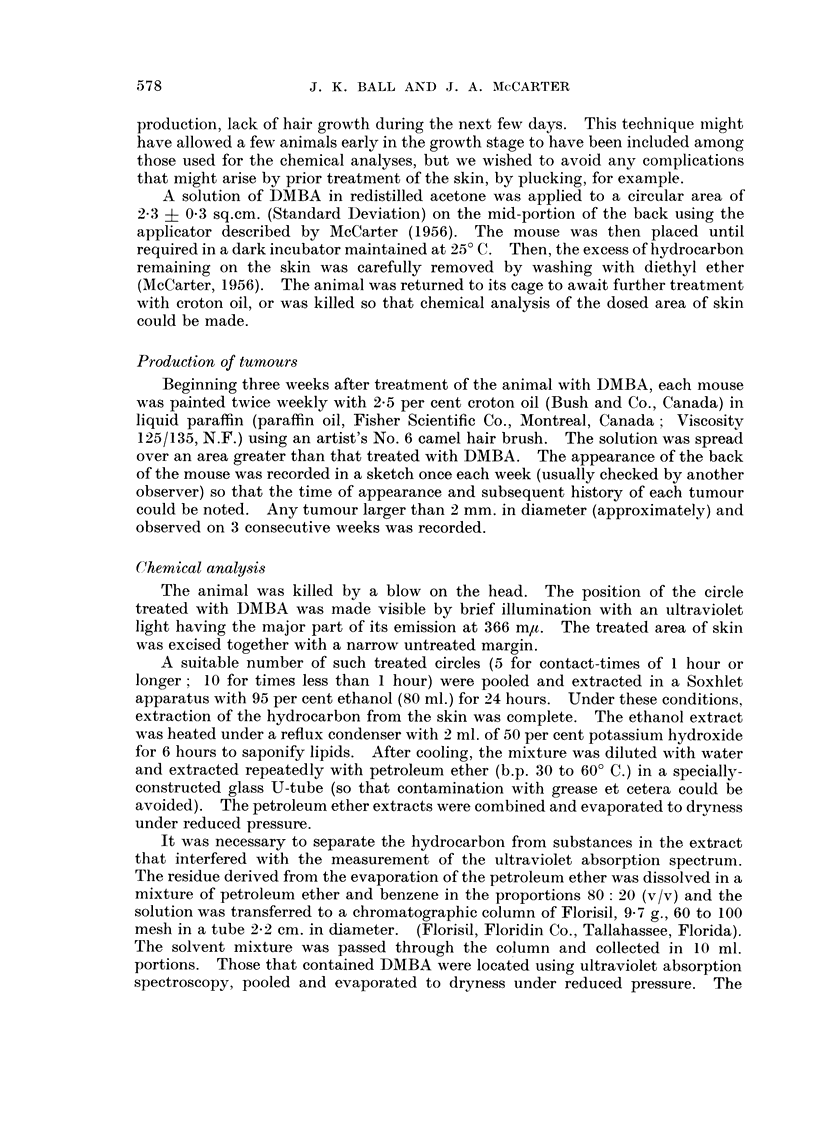

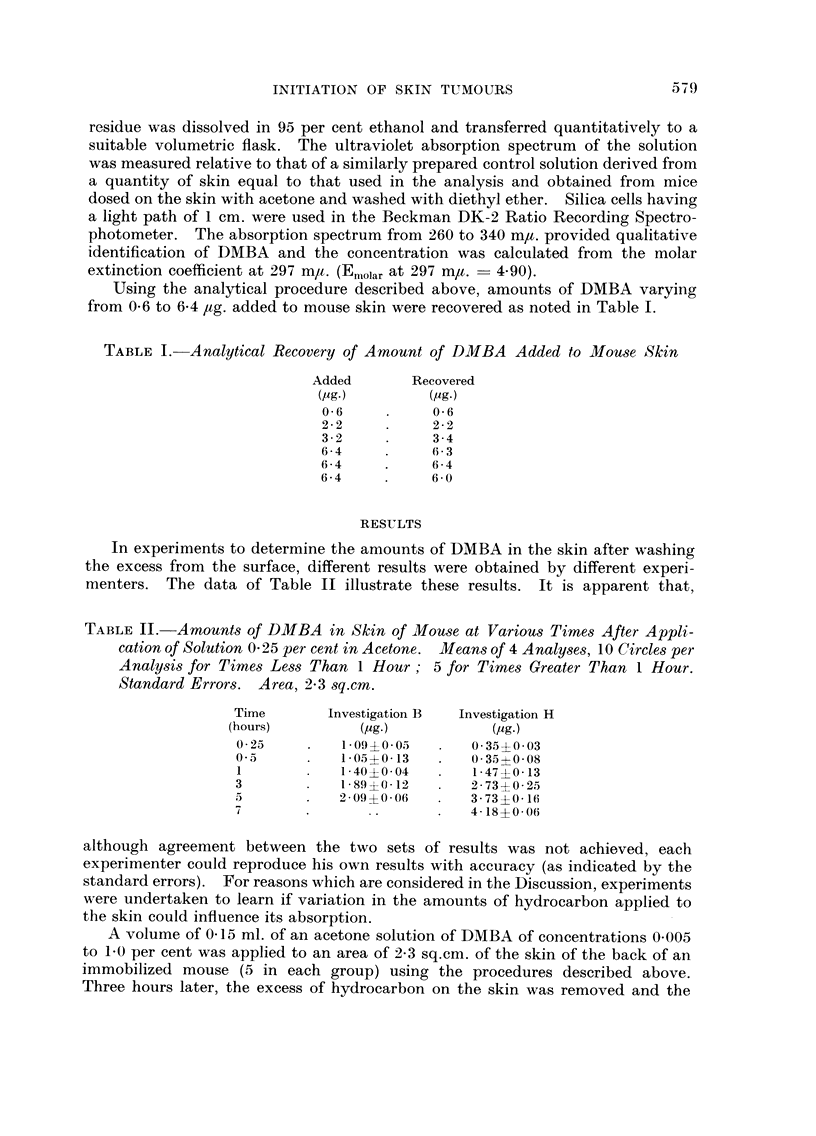

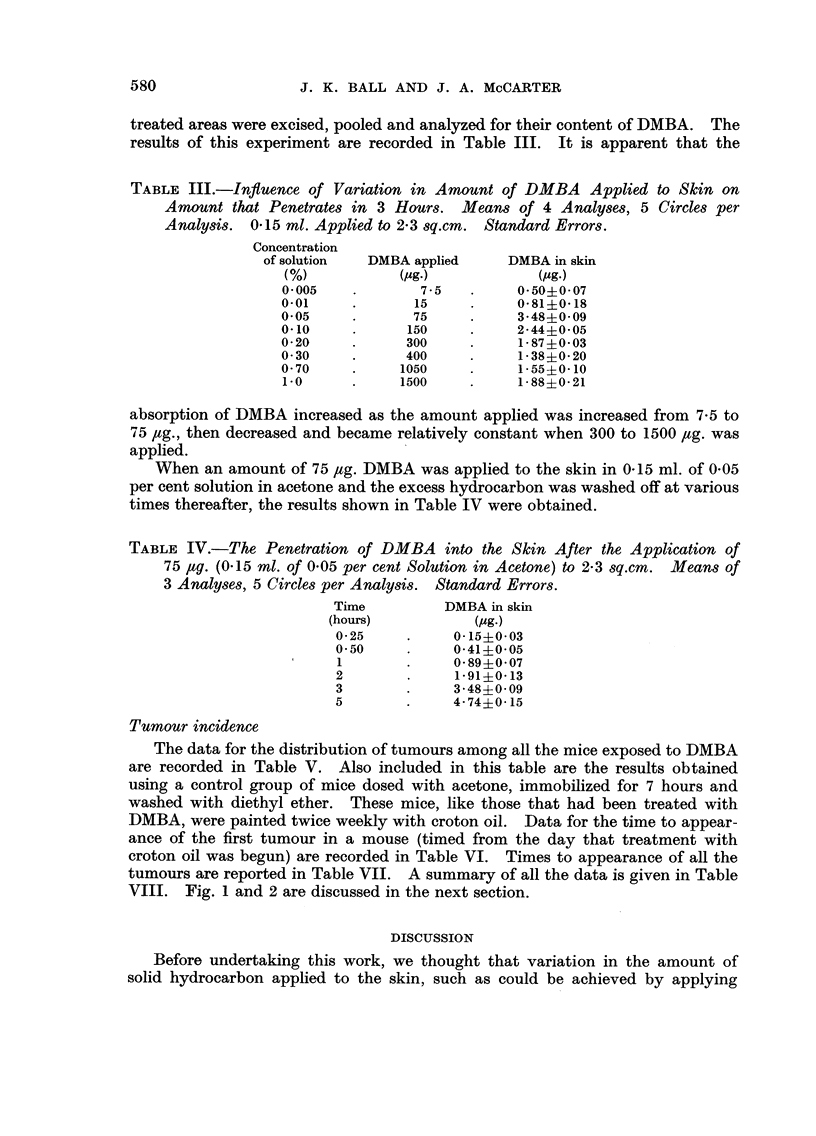

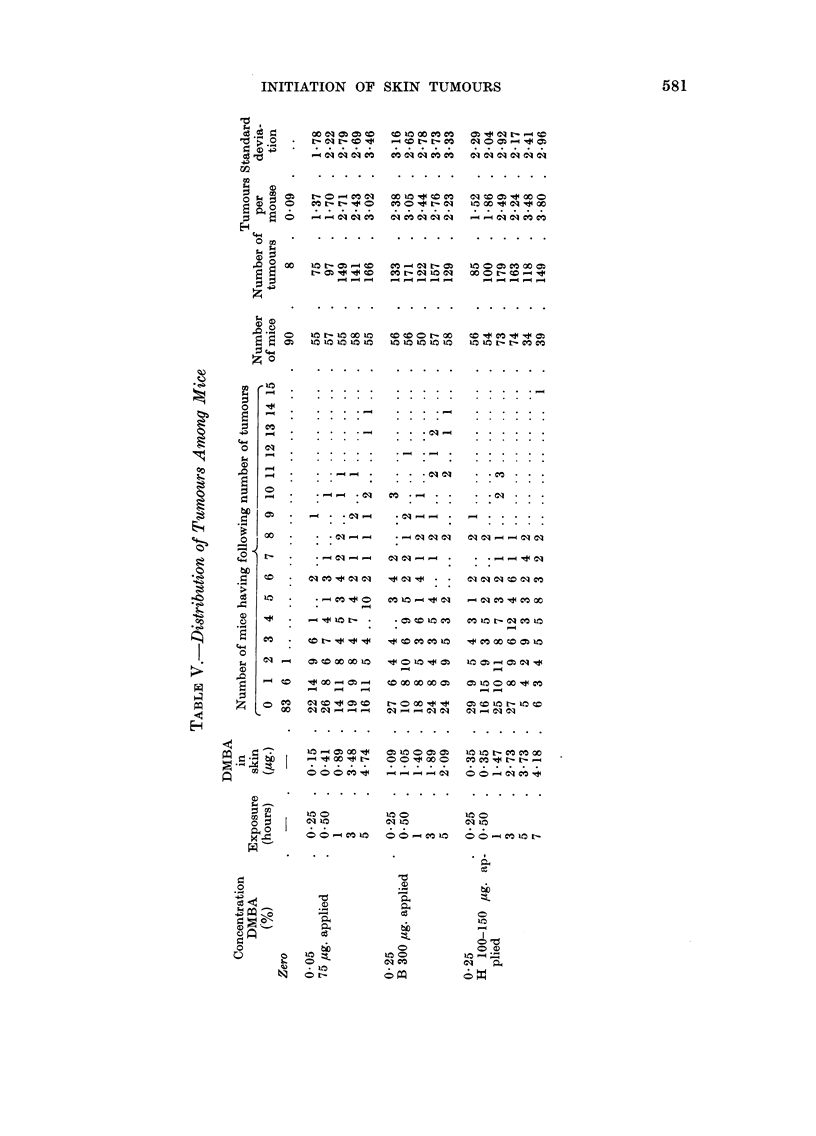

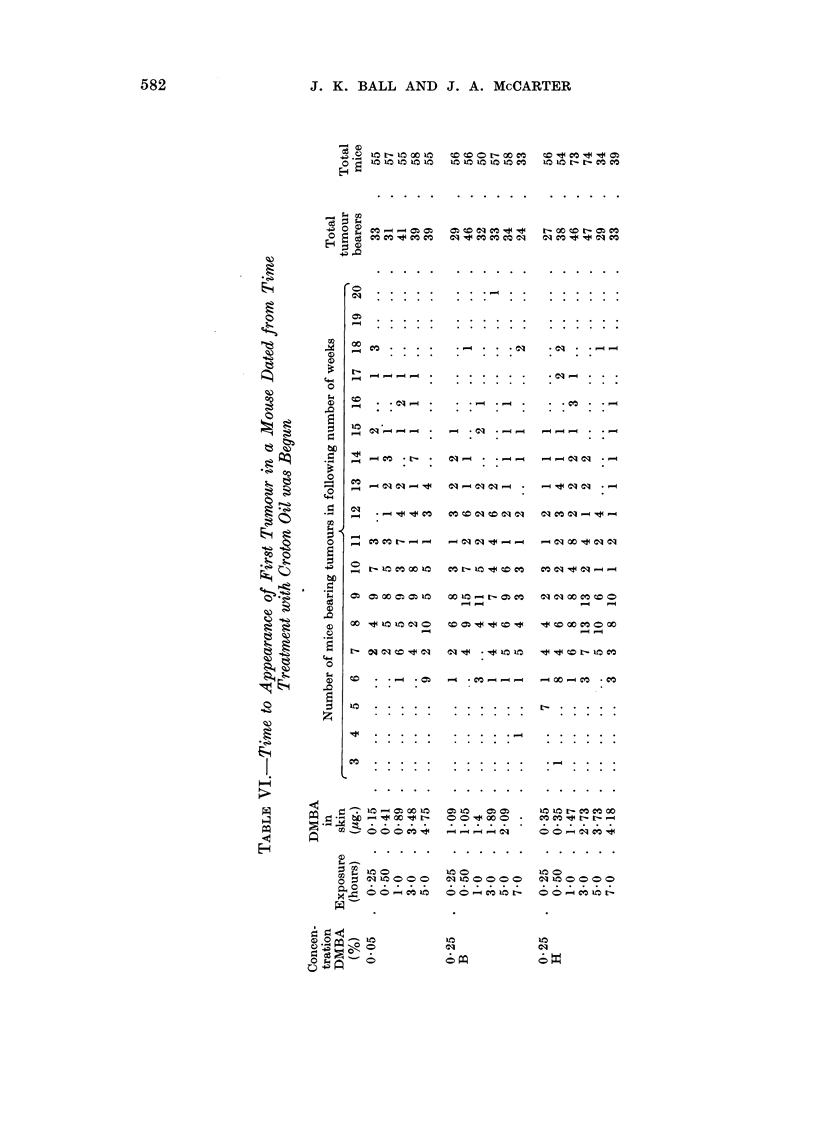

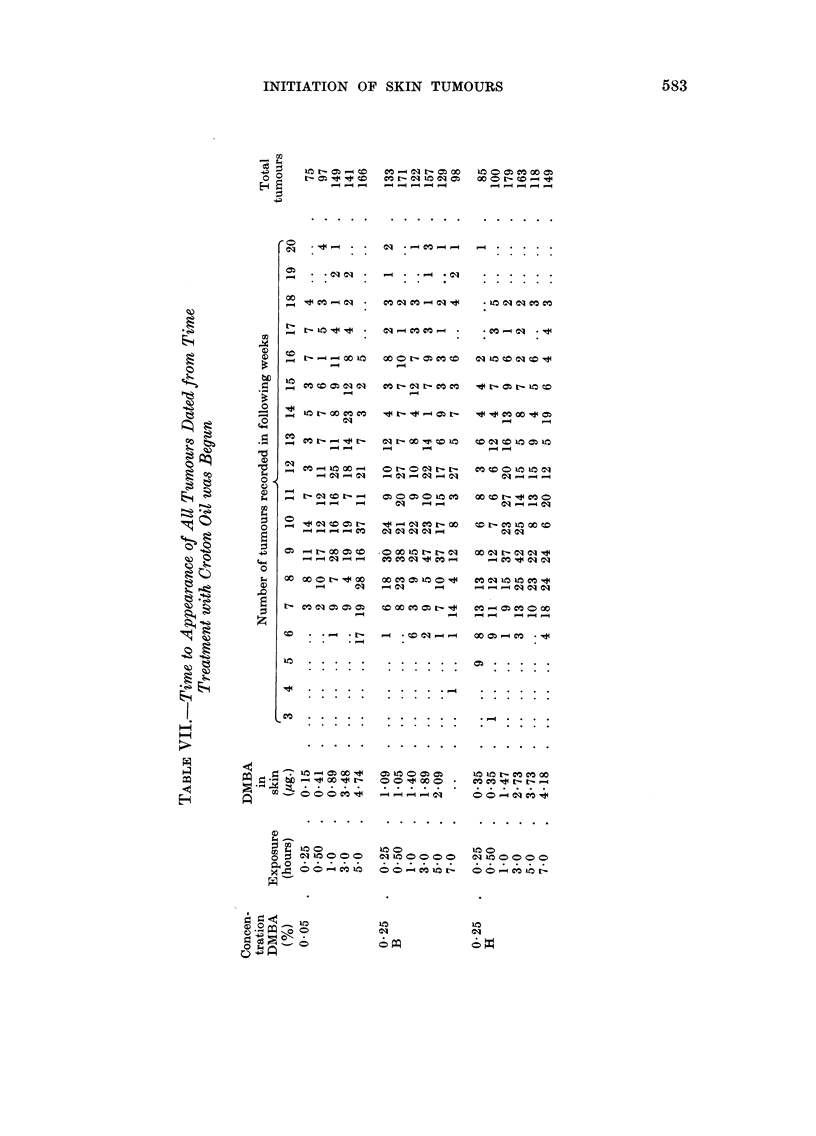

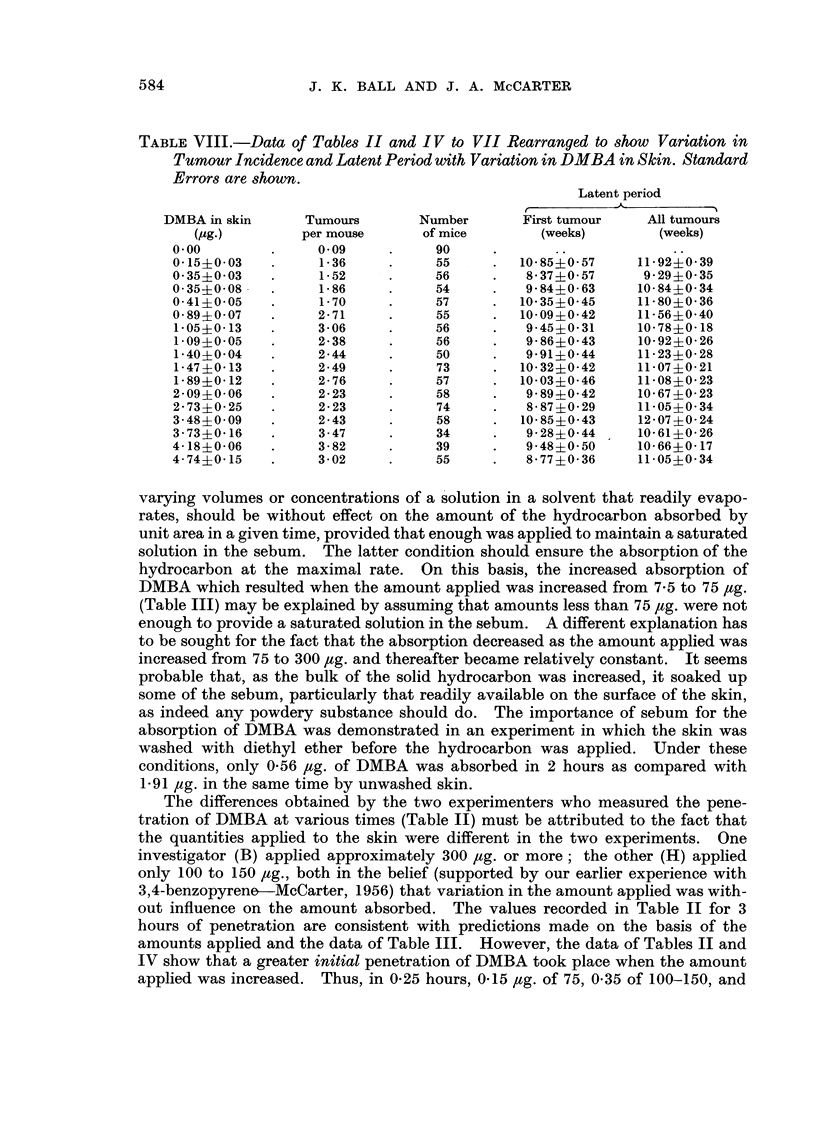

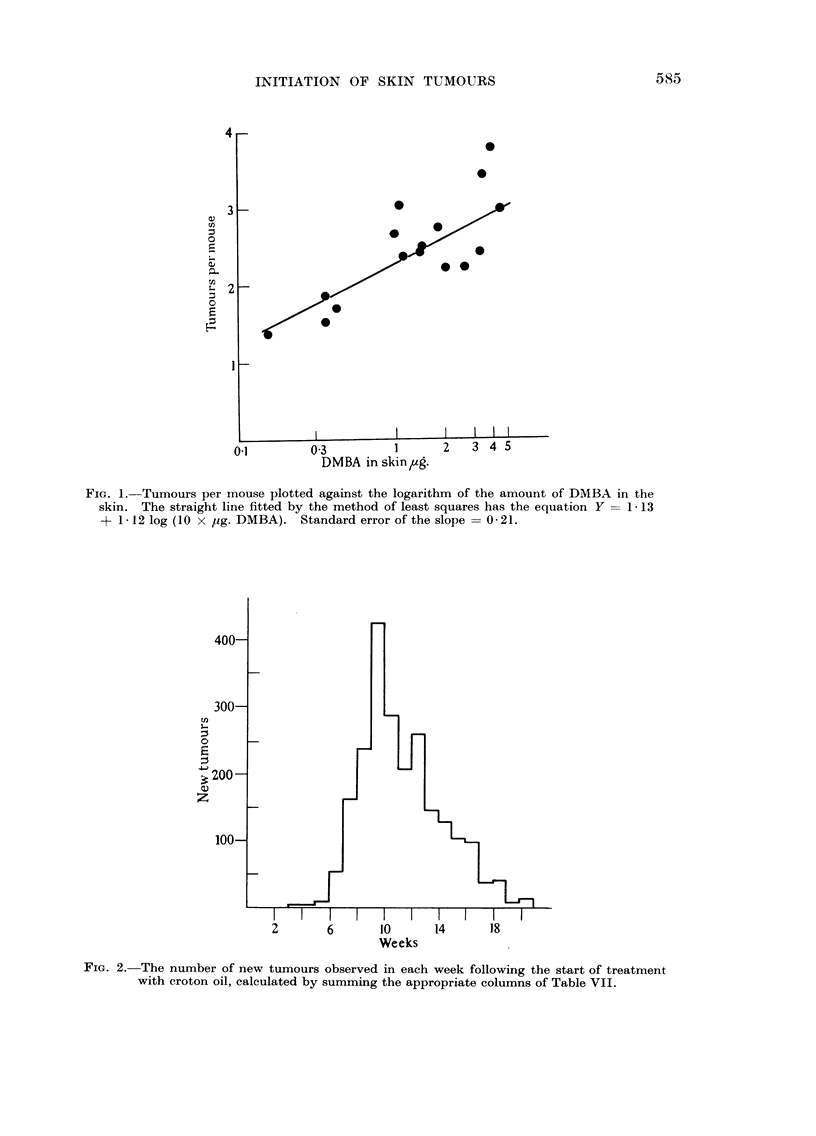

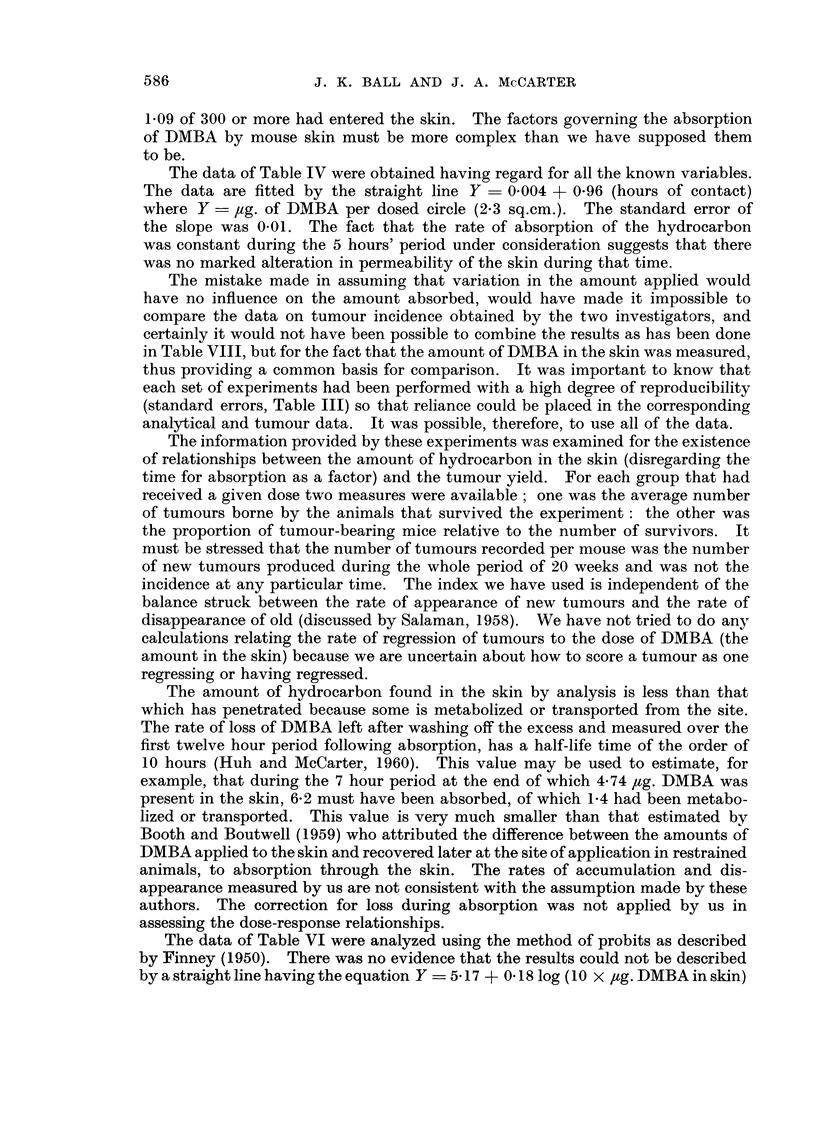

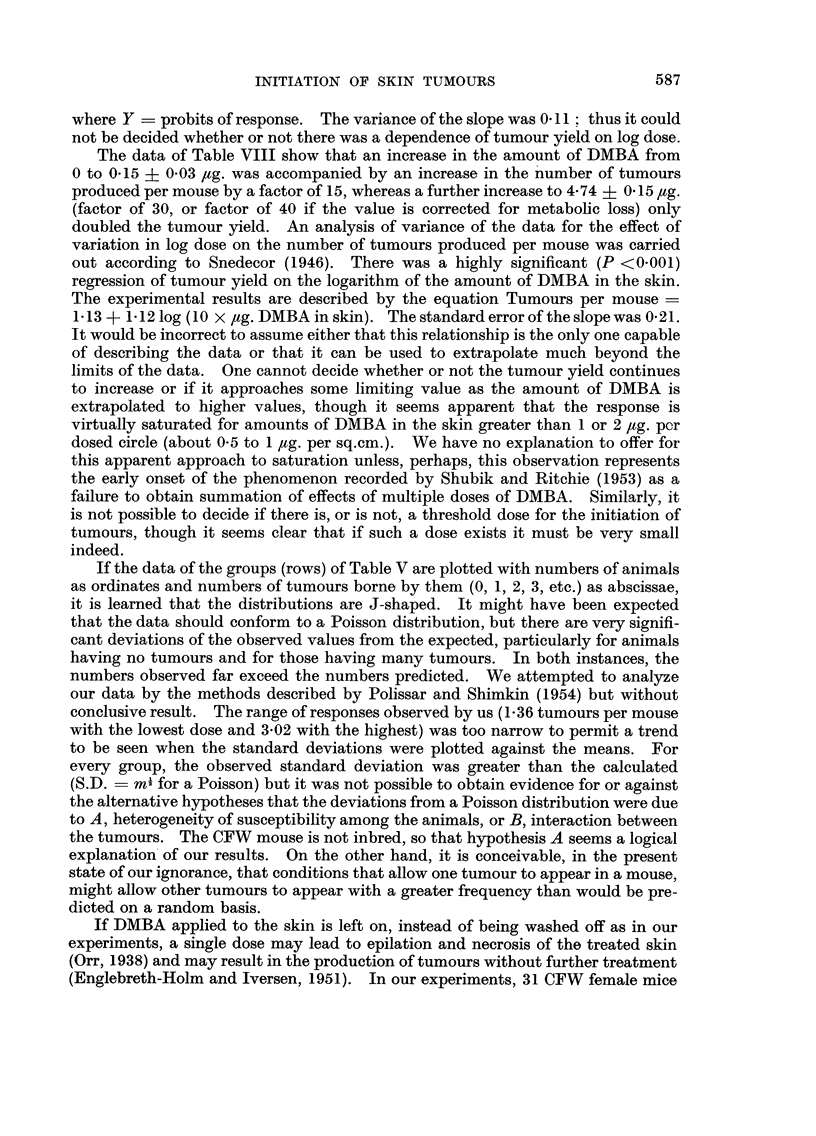

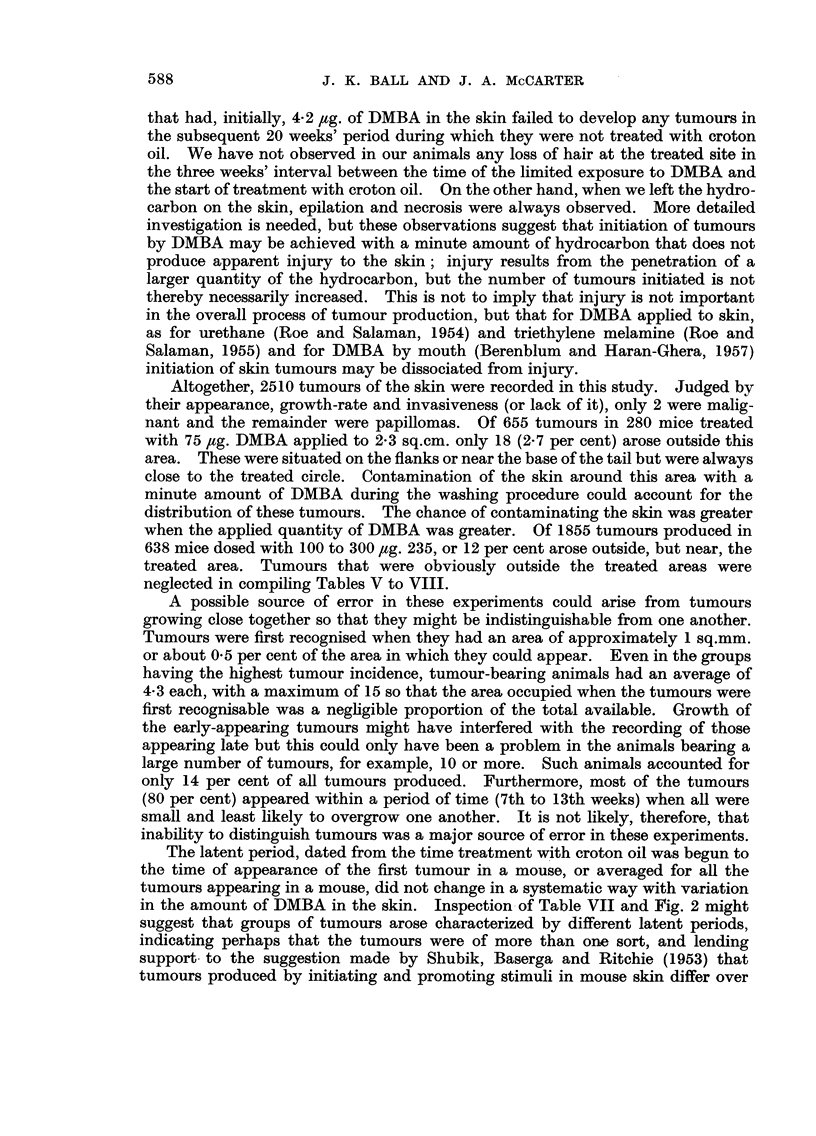

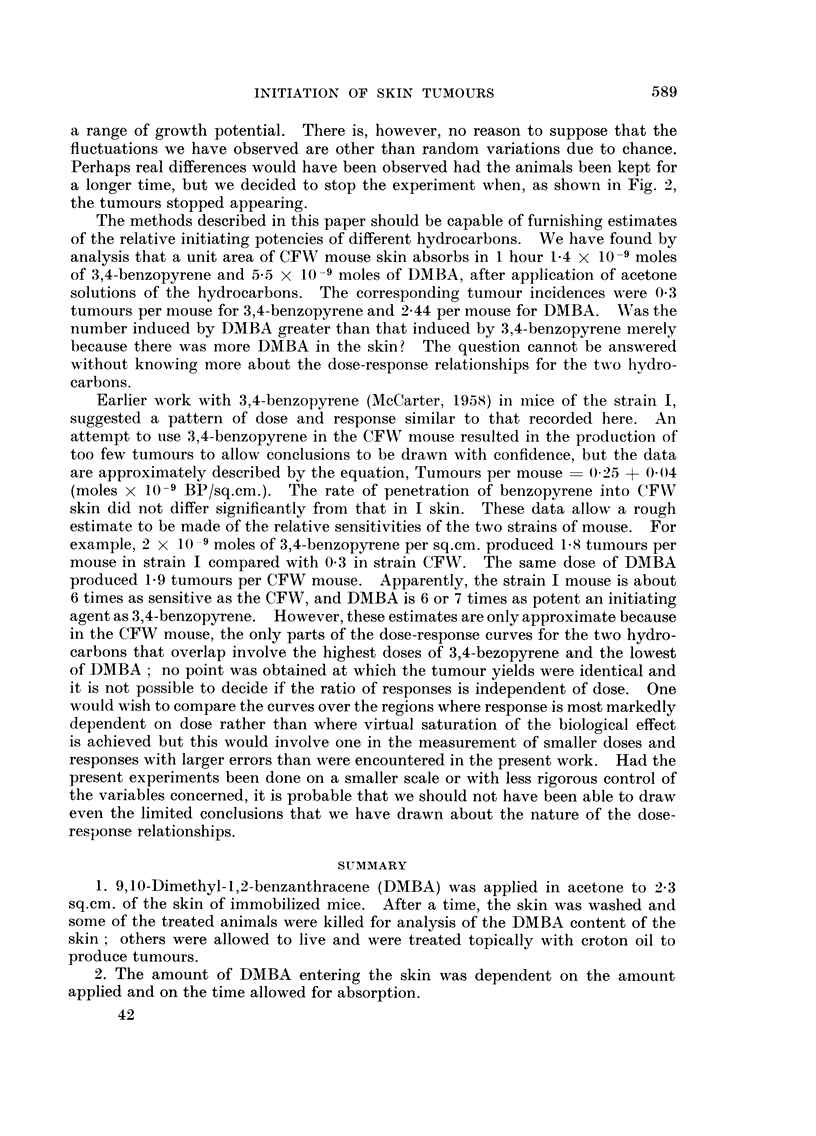

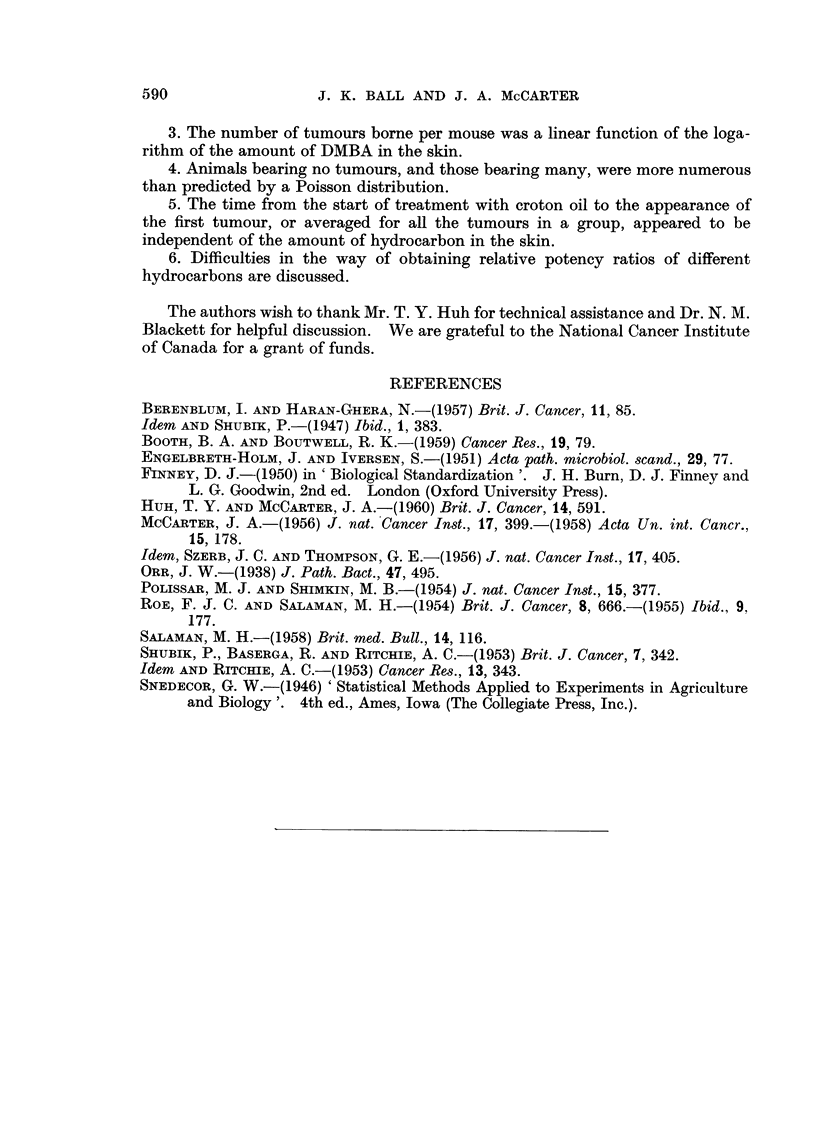


## References

[OCR_01143] MCCARTER J. A., SZERB J. C., THOMPSON G. E. (1956). The influence of area of skin exposed, duration of exposure, and concentration in determining tumor yield in the skin of the mouse after a single application of a carcinogenic hydrocarbon.. J Natl Cancer Inst.

[OCR_01141] MCCARTER J. A. (1956). The control of certain factors of dosage in epidermal carcinogenesis.. J Natl Cancer Inst.

[OCR_01146] POLISSAR M. J., SHIMKIN M. B. (1954). A quantitative interpretation of the distribution of induced pulmonary tumors in mice.. J Natl Cancer Inst.

[OCR_01152] SALAMAN M. H. (1958). Cocarcinogenesis.. Br Med Bull.

[OCR_01154] SHUBIK P., BASERGA R., RITCHIE A. C. (1953). The life and progression of induced skin tumors in mice.. Br J Cancer.

